# Tracking Changes in Neuropathic Pain After Acute Spinal Cord Injury

**DOI:** 10.3389/fneur.2019.00090

**Published:** 2019-02-14

**Authors:** Paulina Simonne Scheuren, Martin Gagné, Catherine Ruth Jutzeler, Jan Rosner, Catherine Mercier, John Lawrence Kipling Kramer

**Affiliations:** ^1^International Collaboration on Repair Discoveries, University of British Columbia, Vancouver, BC, Canada; ^2^Spinal Cord Injury Center, University Hospital Balgrist, University of Zurich, Zurich, Switzerland; ^3^Department of Health Sciences and Technology, Swiss Federal Institute of Technology, Zurich, Switzerland; ^4^The Interdisciplinary Center for Research in Rehabilitation and Social Integration, Quebec, QC, Canada; ^5^School of Kinesiology, University of British Columbia, Vancouver, BC, Canada; ^6^Department of Rehabilitation, Laval University, Quebec, QC, Canada

**Keywords:** neuropathic pain, spinal cord injury, quantitative sensory testing, adaptation, temporal summation, pain modulation

## Abstract

Neuropathic pain represents a primary detrimental outcome of spinal cord injury. A major challenge facing effective management is a lack of surrogate measures to examine the physiology and anatomy of neuropathic pain. To this end, we investigated the relationship between psychophysical responses to tonic heat stimulation and neuropathic pain rating after traumatic spinal cord injury. Subjects provided a continuous rating to 2 min of tonic heat at admission to rehabilitation and again at discharge. Adaptation, temporal summation of pain, and modulation profile (i.e., the relationship between adaptation and temporal summation of pain) were extracted from tonic heat curves for each subject. There was no association between any of the tonic heat outcomes and neuropathic pain severity at admission. The degree of adaptation, the degree of temporal summation of pain, and the modulation profile did not change significantly from admission to discharge. However, changes in modulation profiles between admission and discharge were significantly correlated with changes in neuropathic pain severity (*p* = 0.027; *R*^2^ = 0.323). The modulation profile may represent an effective measure to track changes in neuropathic pain severity from early to later stages of spinal cord injury.

## Introduction

Upwards of 50% of patients with spinal cord injury report signs and symptoms of neuropathic pain (1–4). The impact on the individual and society are enormous, reducing quality of life (5), increasing the risk of other health co-morbidities (6–8), and ultimately driving up health care expenses (9). To ameliorate these negative effects, more effective pain management interventions are urgently needed.

A major problem in developing novel and effective treatments is a lack of quantitative measures to assess changes in physiology that occur alongside fluctuations in the severity of pain. Such measures would be valuable for evaluating biological effects, confirming changes in perception, and increasing the likelihood of translation from basic animal models to the human condition (i.e., phase II clinical trials). While a number of studies have applied quantitative sensory testing in the field of spinal cord injury (10–14), few have been done so longitudinally in a period of fluctuating pain symptoms (e.g., transition from acute to chronic spinal cord injury) (15).

Among emerging techniques to evaluate sensory physiology is the evaluation of tonic heat pain (16). Responses to tonic heat applied for long durations (e.g., 2 min) at noxious temperatures are characterized by an initial period of reduced pain perception followed by increased pain perception. These behavioral changes are associated with discrete peripheral and central processes, which are affected in a variety of chronic pain conditions (i.e., reduced adaptation and increased temporal summation of pain) (10, 11).

The aim of this study was to examine the relationship between behavioral changes to tonic heat and changes in neuropathic pain severity in a cohort of patients with spinal cord injury. Our hypotheses were that (1) neuropathic pain would be associated with diminished adaptation and high temporal summation of pain at admission and (2) changes in adaptation and temporal summation of pain would be associated with changes in neuropathic pain severity between admission and discharge. Tonic heat was examined alongside the severity of neuropathic pain acutely at admission and later at discharge from a rehabilitation center. Adaptation and the temporal summation of pain were extracted from tonic heat curves. The difference between adaptation and the temporal summation of pain was also calculated to reflect the degree of pain modulatory capacity—so-termed modulation profile.

## Materials and Methods

### Subjects

A total of 27 subjects (20 males) with acute spinal cord injury were recruited from the Spinal Cord Injury unit of the Institut de Réadaptation en Déficience Physique de Québec du Centre Intégré Universitaire en Santé et Service Sociaux de la Capitale Nationale. Inclusion criteria were (1) at least 18 years of age and (2) traumatic injury etiology. Exclusion criteria comprised history of neuropathy, chronic pain prior to spinal cord injury, psychiatric disorders, and cognitive deficits. This study was carried out in accordance with the recommendations of the institutional review board of the Institut de Réadaptation en Déficience Physique de Québec. The protocol was approved by the institutional review board of the Institut de Réadaptation en Déficience Physique de Québec (ref. number: 2011-258). All subjects gave written informed consent in accordance with the Declaration of Helsinki.

### Clinical Assessments of Injury and Pain Characteristics

Within the first 3 weeks of admission to the rehabilitation center, the severity and neurological level of spinal cord injury were assessed according to International Standards for the Neurological Classification of Spinal Cord Injury (ISNCSCI) (17). Briefly, two somatosensory modalities (i.e., mechanosensation and nociception) were semi-quantitatively examined in 28 dermatomes according to ISNCSCI using light touch and pinprick testing, respectively.

The presence of neuropathic and/or musculoskeletal pain was assessed in a clinical examination according to the standardized International Spinal Cord Injury Pain Basic Data Set (ISCIPBDS) (18). The ISCIPBDS questionnaire was developed to standardize the collection and reporting of pain in the spinal cord injury population. Specifically, subjects were interviewed to determine the location of pain (i.e., 8 predefined areas), type of pain (neuropathic [below or at level] or nociceptive [musculoskeletal, visceral, other]), pain intensity (average pain), temporal pattern (onset of pain, duration of pain, pattern of pain occurrence), and pain interference with daily living activities. Pain intensity was rated on an 11-point scale from 0 (“no pain”) to 10 (“pain as bad as you can imagine”) and pain interference with a 7-point scale from 0 (“not at all”) to 6 (“very much”). A trained physiatrist of the spinal cord injury unit conducted the pain assessments (i.e., ISCIPBDS) at both admission and discharge.

### Tonic Heat Pain Testing

**Figure 1:**
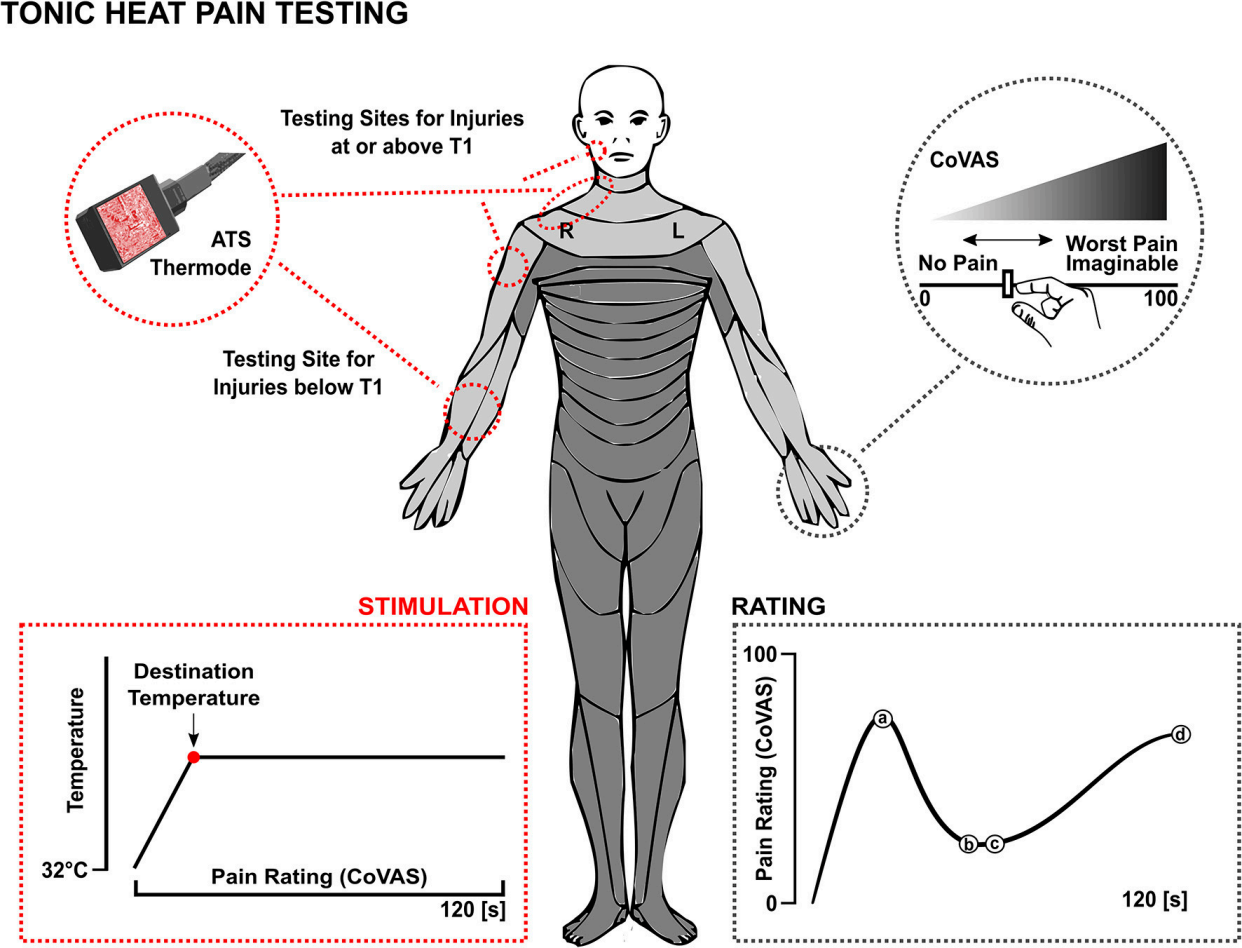
Tonic heat pain testing protocol. ATS, advanced thermal stimulator; CoVAS, continuous visual analog scale; T1, first thoracic vertebra.

A trained examiner performed the admission and discharge evaluation of tonic heat. Testing was performed at least two dermatomes above the level of injury to ensure that an area with intact sensory function was being examined. Subjects with lesion levels below T1 were tested on the volar surface of the forearm (Figure 1). Subjects with lesion levels at or above T1 were tested at least two dermatomes above the lesion level (i.e., intact sensation). The testing site was identical for each subject at both time points, which ensured repeated measures for each subject. All subjects were physically able to manipulate the computerized visual analog scale (CoVAS, described in more detail below) to rate their perceived pain over time.

The Pathway Pain and Sensory Evaluation System (Advanced Medical Systems, Durham, U.S.) was used to deliver individual heat pain thresholds and perform the tonic heat pain testing. This was done with the advanced thermal stimulator (ATS) thermode (Figure 1). The activation area of the contact probe was 30 x 30 mm. The stimulator was held on the skin by the examiner.

The tonic heat pain testing protocol is illustrated in Figure 1. To account for differences in individual pain thresholds, the temperature rose from the baseline (32°C) to an individually predetermined destination temperature, which was intended to evoke a perceived intensity of 50 out of 100 on the CoVAS (left edge = no pain, 0, right edge = worst pain imaginable, 100). The thermode ramped up to the destination temperature and remained constant for 2 min. During these 2 min, subjects were instructed to continuously evaluate their perceived pain using the CoVAS.

### Data Analysis

The degree of adaptation and temporal summation of pain were extracted from each tonic heat curve. Adaptation was determined as the initial decrease in pain perception after the ATS thermode reached peak temperature. Subsequent increases in pain perception were determined as temporal summation of pain. The start of the adaptation phase was set at the highest pain rating after the peak pain rating. The end of the adaptation phase was set at the following lowest pain rating. The start of the temporal summation of pain phase was set at the first increase in pain rating following the adaptation phase. If there was no adaptation phase, the start of the temporal summation of pain phase was set after the destination temperature was reached. The end of the temporal summation of pain phase was set at the end of the increase in pain ratings. The degree of adaptation was calculated by subtracting the lowest pain rating (b) from the highest (a) of the adaptation phase (Figure 1; degree of adaptation = ΔADT = a − b). The degree of temporal summation of pain was calculated by subtracting the lowest pain rating (c) from the highest (d) of the temporal summation phase (Figure 1; degree of temporal summation of pain = ΔTSP = d − c). A novel tonic heat outcome, namely the modulation profile was extracted, which represented the difference between the degree of adaptation and that of temporal summation of pain [ΔADT - ΔTSP = (a − b) − (d − c)] (Figure 1). This measure was intended to quantify the extent to which the degree of adaptation was higher or lower compared to that of temporal summation of pain for a given tonic heat curve.

### Statistical Analysis

Statistical analyses were performed using the R computing environment version 1.0.143 for Mac OS X. All data were tested for normal distribution using the Kolmogorov-Smirnov test. Bonferroni correction was used to account for multiple comparisons. Statistical significance was set at α = 0.05.

The statistical analysis comprised two main tests. The difference between admission and discharge tonic heat outcomes was tested using the Mann-Whitney *U*-test. Linear regression analysis was applied to examine the relationship between (a) neuropathic pain and tonic heat outcomes at admission and (b) changes in neuropathic pain and tonic heat outcomes during the transition from admission to discharge. The relationship between musculoskeletal pain and tonic heat outcomes was also analyzed using linear regression. All linear models were adjusted for the length of stay in rehabilitation.

## Results

### Subjects

**Figure 2:**
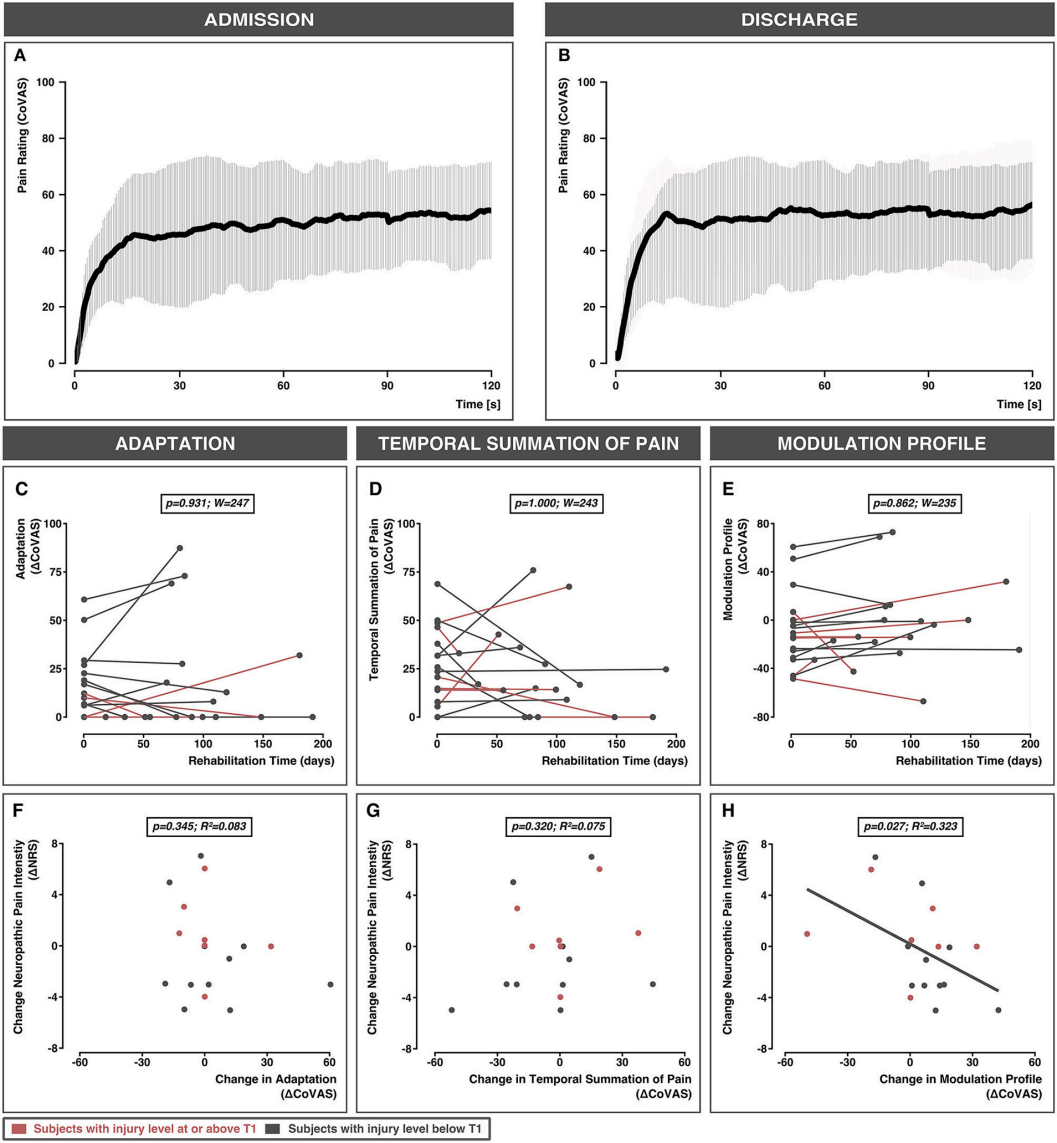
Averaged pain ratings over the 120 s testing period are shown for all 18 subjects with complete data at admission **(A)** and discharge **(B)**. The gray bars represent standard deviations. The effect of time on adaptation **(C)**, temporal summation of pain **(D)**, and modulation profile **(E)** are shown for individual rehabilitation times. Bottom panel: The relationship between the change in neuropathic pain intensity and change in adaptation **(F)**, change in temporal summation of pain **(G)**, and change in modulation profile **(H)**. Subjects with injury levels at or above T1 are colored in red. Subjects with injury levels below T1 are colored in gray. CoVAS, continuous visual analog scale; NRS, numeric rating scale.

All 27 subjects underwent tonic heat pain testing at admission. Nine subjects did not participate in the tonic heat pain testing at discharge. Reasons for dropout included technical problems (*n* = 1), development of hypersensitivity to heat (*n* = 1), subject not available on discharge (*n* = 6), and death (*n* = 1). The 18 subjects examined at discharge included 12 males. The demographic and spinal cord injury characteristics are shown in Table 1. Neuropathic pain severity and tonic heat outcomes at admission and discharge are shown in Tables 2A,B, respectively. The average tonic heat curve over all subjects at admission and discharge is shown in Figures 2A,B, respectively.

**Table 1:** Demographic and clinical characteristics.

**Subjects**	**Age group [years]**	**AIS (A–D)**	**Etiology**	**SCI NLI**	**Time since injury at admission [days]**	**Duration of rehabilitation [days]**
1	18–25	B	Other	T7	54	80
2	26–30	D	MVA	C5	43	180
3	36–40	A	MVA	T11	24	82
4	51–55	A	Fall	T2	32	191
5	41–45	D	MVA	C5	53	148
6	31–35	B	MVA	C7	55	110
7	31–35	A	Other	T12	19	90
8	71–75	D	Other	C5	26	18
9	31–35	B	MVA	C5	42	99
10	18–25	B	Other	L2	23	73
11	36–40	B	MVA	L3	26	119
12	51–55	D	Fall	C4	50	51
13	18–25	A	MVA	T5	23	69
14	51–55	C	Other	T11	45	77
15	26–30	A	MVA	T6	34	84
16	31–35	B	Other	L3	29	108
17	41–45	D	MVA	T11	27	34
18	61–65	D	Fall	C2	19	55
19	18–25	B	Fall	C5	70	/
20	18–25	D	Other	T5	38	/
21	41–45	C	Other	T11	41	/
22	56–60	B	Fall	T12	35	/
23	31–35	B	Other	T10	37	/
24	26–30	A	Other	T1	31	/
25	46–50	D	Other	L2	28	/
26	56–60	D	Fall	C7	36	/
27	41–45	A	Other	T11	31	/
Median (IQR)	36 (30.5–50.5)				34 (26.5–42.5)	83 (70–109.5)

*AIS, American Spinal Cord Injury Association Impairment Scale; A, no sensory or motor function is preserved; B, sensory function is preserved below the level of the injury, but there is no motor function; C, motor function is preserved below the neurological level, and more than half of the key muscles below the neurological level have a muscle grade of < 3; D, motor function is preserved below the neurological level, and at least half of the key muscles below the neurological level have a muscle grade of ≥ 3; C, cervical; F, female; IQR, interquartile range; L, lumbar; M, male; MVA, motor vehicle accident; NLI, neurological level of injury; SCI, spinal cord injury; T, thoracic; yrs, years*.

**Table 2:** Study findings.

**A) Admission**							**B) Discharge**					
**Subject**	**Neuropathic pain (NRS)**	**Adaptation (ΔCoVAS)**	**Temporal summation of pain (ΔCoVAS)**	**Modulation profile (ΔCoVAS)**	**Average pain rating (CoVAS)**	**Test temperature (**°**C)**	**Neuropathic pain (NRS)**	**Adaptation (ΔCoVAS)**	**Temporal summation of pain (ΔCoVAS)**	**Modulation profile (ΔCoVAS)**	**Average pain rating (CoVAS)**	**Test temperature (**°**C)**
1	3	26.9	31.7	−4.8	74.5	47.9	0	87.3	75.9	11.4	61.8	46.3
2	0	0	0	0	83.9	49.1	0	31.9	0	31.9	33.9	49
3	2	29.3	0	29.3	44.8	46	9	27.5	14.9	12.6	46	45
4	0	0	23.6	−23.6	26.9	46	0	0	24.7	−24.7	57.8	47
5	0	9.9	20.7	−10.8	41.1	48	3	0	0	0	48.2	48.5
6	0	0	48.7	−48.7	47.7	48.6	6	0	67.4	−67.4	47.9	47.6
7	3	16.9	50	−33.1	32.9	47.9	8	0	27.4	−27.4	69.6	–
8	4	0	46.5	−46.5	52.2	47	4	0	33	−33	22.4	47.5
9	4.5	0	14.8	−14.8	41.3	48.5	5	0	14.2	−14.2	65.2	48.5
10	0	50.2	0	50.2	73.9	48	0	69	0	69	36	49
11	5	22.6	68.8	−46.2	45.5	–	0	12.8	16.7	−3.9	45.8	46.5
12	6	12.2	5.5	6.7	28.7	47	7	0	42.7	−42.7	72.2	48
13	1	6	31.8	−25.8	38.4	48	0	17.8	36	−18.2	41.8	48
14	7	19	25.8	−6.8	32	48	4	0	0	0	51.4	48
15	9	60.7	0	60.7	57.5	46.5	4	72.9	0	72.9	44.5	46
16	3	6.1	7.9	−1.8	38.4	47	0	8	9	−1	59.8	–
17	7	6.7	37.9	−31.2	45.6	47.4	4	0	17	−17	53.2	46.8
18	4	0	14	−14	49.2	46.5	0	0	13.9	−13.9	54.9	47
19	5	51.1	19	32.1	39.7	45.5	–	–	–	–	–	–
20	0	40.4	33.9	6.5	59.8	47.7	–	–	–	–	–	–
21	4	0	0	0	65.4	47.5	–	–	–	–	–	–
22	0	0	0	0	82.2	46.7	–	–	–	–	–	–
23	0	0	55.1	−55.1	44.4	48	–	–	–	–	–	–
24	4	6	0	6	40.4	47	–	–	–	–	–	–
25	10	0	17.9	−17.9	26.8	46.8	–	–	–	–	–	–
26	5	0	9	−9	49.5	46	–	–	–	–	–	–
27	0	0	0	0	51.2	47	–	–	–	–	–	–
Median (IQR)	3.0 (0.0–5.0)	6.0 (0.0–20.8)	17.9 (0.0–32.9)	−6.8 (−24.7–3.0)	45.5 (39.1–48.7)	47.2 (46.7–48)	3.5 (0.0–4.8)	0.0 (0.0–25.1)	15.8 (2.3–31.6)	−8.9 (−23–8.6)	49.8 (44.8–59.3)	47.6 (46.7–48.1)

*A) At admission and B) at discharge. CoVAS, continuous visual analog scale; NRS, numeric rating scale; IQR, interquartile range*.

### Main Effect of Time: Test Temperature, Average Pain Rating, and Tonic Heat Outcomes

The 2-min test temperature did not change significantly between admission and discharge (W = 118, *p* = 0.611). The average pain rating during the 2-min test period did not change from admission to discharge (W = 196, *p* = 0.319). Neither the degree of adaptation (W = 247, *p* = 0.931), temporal summation of pain (W = 243, *p* = 1.000), nor the modulation profile (W = 235, *p* = 0.862) significantly changed from admission to discharge (Figures 2C–E).

### Relationship Between Neuropathic Pain and Tonic Heat Outcomes at Admission

At admission, adaptation (*p* = 0.395; *R*^2^ = 0.029), temporal summation of pain (*p* = 0.871; *R*^2^ = 0.001), and the modulation profile (*p* = 0.506; *R*^2^ = 0.018) were not significantly associated with neuropathic pain (Supplementary Table 1).

### Relationship Between Changes in Neuropathic Pain and Changes in Tonic Heat Outcomes From Admission to Discharge

Changes in adaptation or temporal summation of pain were not related to changes in neuropathic pain severity from admission to discharge (adaptation: *p* = 0.345, *R*^2^ = 0.083; temporal summation: *p* = 0.320, *R*^2^ = 0.075) (Figures 2F,G). However, changes in neuropathic pain severity were significantly associated with the changes in modulation profiles (*p* = 0.027; *R*^2^ = 0.323) (Figure 2H).

### Relationship Between Changes in Musculoskeletal Pain and Changes in Tonic Heat Outcomes From Admission to Discharge

No relationship was found between changes in musculoskeletal pain and changes in tonic heat outcomes (adaptation: *p* = 0.418, *R*^2^ = 0.160; temporal summation: *p* = 0.682, *R*^2^ = 0.013; modulation profile: *p* = 0.364, *R*^2^ = 0.064) from admission to discharge.

## Discussion

The aim of the present study was to examine changes in behavioral responses to tonic heat alongside fluctuations in the severity of neuropathic pain after spinal cord injury. Our primary finding was that changes in the severity of neuropathic pain corresponded with changes in the modulation profile. This occurred such that subjects with increasing pain exhibited less adaptation and/or more temporal summation, whereas subjects with decreasing pain experienced more adaptation and/or less temporal summation. Overall, these observations suggest that tonic heat stimulation is a valuable quantitative method to objectively track changes related to the severity of neuropathic pain in patients with spinal cord injury.

Our approach to examine adaptation and temporal summation of pain using tonic heat is similar to previous studies. Applying a 30 s constant heat stimulus, Albu and colleagues observed significantly decreased adaptation in patients with chronic spinal cord injury compared to healthy controls, but no differences between patients with and without neuropathic pain (11). Gruener and colleagues reported similar observations in terms of adaptation but significantly higher temporal summation of pain in patients with neuropathic pain compared to those without neuropathic pain and healthy controls (10). All of the aforementioned observations, in addition to others applying quantitative sensory testing techniques (10, 11, 13, 19, 20), have been limited to cross-sectional investigations in chronically injured patients.

In the present study, neither increased temporal summation of pain nor decreased adaptation was associated with the severity of neuropathic pain after spinal cord injury. Additionally, changes from admission to discharge in either parameter alone were not sufficient to track fluctuations in neuropathic pain ratings over time. Discrepancies with previous studies (10, 11) could be attributable to differences in tonic heat stimulation techniques. For example, we performed a longer tonic heat stimulus (2 min) delivered at a moderate pain intensity (CoVAS = 50 units), extracting adaptation and temporal summation of pain from a single tonic heat profile. In comparison, Gruener and colleagues utilized shorter durations of pain and different intensities to separately evaluate adaptation and temporal summation (10).

We did observe, however, that the difference between the amount of adaptation and that of temporal summation of pain, termed the modulation profile, correlated with fluctuations in neuropathic pain severity. Importantly, this relationship was independent of length of rehabilitation, which was included as a covariate in the linear regression analysis. There was no such correlation with musculoskeletal pain, suggesting a specific effect of neuropathic pain. The relationship between modulation profile and neuropathic pain intensity was evidenced as (1) decreases corresponding with worsening pain symptoms (i.e., decreased adaptation, increased temporal summation of pain, or both), (2) increases corresponding with decreasing neuropathic pain intensity (i.e., increased adaptation, decreased temporal summation of pain, or both), and (3) relative stability among subjects with no change in neuropathic pain intensity. To our knowledge, this is the first study to employ such a metric as a means to quantify changes in response to tonic heat stimulation.

That an aggregate of adaptation and temporal summation better reflects underlying changes in nociception associated with fluctuations in neuropathic pain compared to either measure alone is intuitive. The conventional approach of examining these outcomes as separate events is problematic because it inherently assumes that chronic pain can be characterized by a single outcome. More likely is a situation where a variety of changes in nociception accompany chronic neuropathic pain. These may be, in part, dependent on pre-existing sensory profiles (i.e., before spinal cord injury). Previous studies indicate considerable heterogeneity in tonic heat pain profiles in healthy subjects, with substantial proportions of people at both extremes—some demonstrating only adaptation, others only temporal summation (21). These naturally occurring “outliers,” in turn, could contribute to differences in how some subjects' profiles change in response to persisting, resolving, or worsening neuropathic pain.

The question then becomes what pathophysiological mechanisms associated with neuropathic pain underlie changes in tonic heat profiles? One possibility is impaired endogenous descending inhibitory control. This has been widely reported across a variety of chronic pain conditions (22, 23), including spinal cord injury (10, 11). In a healthy subject, descending inhibition can be activated by a long duration noxious stimulus (24). In the absence of descending inhibition (or presence of severe impairment), pain ratings to tonic heat become amplified in otherwise normal appearing dermatomes. This is evidenced as a reduction in anti-nociception (i.e., adaptation) and/or increased pro-nociception (i.e., temporal summation of pain). Further longitudinal studies incorporating a test of descending inhibitory control (e.g., conditioned pain modulation) in patients with spinal cord injury are needed to further address this theory.

Overall, there has been a lack of longitudinal studies that incorporate quantitative sensory testing techniques to examine changes from acute to more chronic time-points after spinal cord injury. This can likely be attributed to the difficulty evaluating neuropathic pain and performing quantitative testing in the very acute stages of traumatic injury. To this end, our study has provided valuable insights. However, there are limitations that warrant discussion. First, the total sample size is small. Nine subjects were lost to follow up examination of tonic heat at discharge, decreasing our original sample from 27 to 18. Acute traumatic spinal cord injury is a relatively rare event and low recruitment is a common problem. Nevertheless, 18 subjects is similar in size to comparable published longitudinal studies (15). Second, and related to sample size, we did not examine differences in tonic heat outcomes between at- and below level neuropathic pain. Different types of neuropathic pain likely have different underlying mechanisms (25, 26), and thus may yield differential sensory outcomes. Last, our analysis did not take into account medications, which could have independently impacted adaptation and temporal summation of pain (27, 28). Accounting for individual medication profiles or previous toxin exposure was not feasible given the small sample size, nor was withholding medications. However, medications were kept stable over time for each subject, to ensure that they did not influence the change in tonic heat profiles over time.

## Conclusion

In summary, changes in neuropathic pain occurring during the transition from early to late spinal cord injury can be tracked using a 2-min tonic heat paradigm. The modulation profile, an aggregate of adaptation and temporal summation of pain, was demonstrated to be a valuable metric to quantify changes in response to tonic heat stimulation. Additional studies are warranted to investigate the application of such modulation profiles as an objective outcome to evaluate interventions aimed at relieving chronic neuropathic pain.

## Author Contributions

PS contributed substantially to the study conception, data analysis and interpretation. Furthermore, she drafted the research article. MG was involved in the data collection and revised the research article. CJ was substantially involved in the data analysis, and interpretation, and revised the research article. JR was involved in the interpretation of the data and revised the research article. CM made substantial contributions to the study conception and design and revised the research article. JK contributed substantially to the study conception, and was involved in the data analysis and interpretation, and was involved in drafting the manuscript.

### Conflict of Interest Statement

The authors declare that the research was conducted in the absence of any commercial or financial relationships that could be construed as a potential conflict of interest.

## Supplementary Material

Supplementary Table 1 Linear model statistics for the relationship between neuropathic pain intensity and tonic heat outcomes.
