# Supplementary material for: Tracking Changes in Neuropathic Pain After Acute Spinal Cord Injury
Source: Front Neurol. 2019 Feb 14;10:90. doi: 10.3389/fneur.2019.00090 (PMC6382744; doi:10.3389/fneur.2019.00090)
Supplement: Supplementary Table 1 — Linear model statistics for the relationship between neuropathic pain intensity and tonic heat outcomes. [file Table_1.DOCX]

Supplementary Material

Tracking Changes in Neuropathic Pain after Acute Spinal Cord Injury

Paulina Simonne Scheuren, Martin Gagné, Catherine Ruth Jutzeler, Jan Rosner, Catherine Mercier, John Lawrence Kipling Kramer*

*** Correspondence:** Corresponding Author: kramer@icord.org

# Supplementary Figures and Tables

| **Supplementary Table 1:**  **Linear models: Relationship between neuropathic pain and tonic heat outcomes** | | | | | |
| --- | --- | --- | --- | --- | --- |
| ***Neuropathic Pain (admission)*** | ***Coefficient (ß)*** | ***SE*** | ***P-value*** | ***R^2^*** | ***F-statistic*** |
| Model 1a |  |  |  |  |  |
| Intercept | 2.83091 | 0.71829 | 0.000576 |  |  |
| Adaptation (admission) | 0.02765 | 0.03202 | 0.395997 | 0.02897 | 0.7459 |
| Model 1b |  |  |  |  |  |
| Intercept | 3.304522 | 0.844966 | 0.000623 |  |  |
| Temporal summation of pain (admission) | -0.004838 | 0.029384 | 0.870535 | 0.001083 | 0.02711 |
| Model 1c |  |  |  |  |  |
| Intercept | 3.30695 | 0.59737 | 9.38e-06 |  |  |
| Modulation Profile (admission) | 0.01404 | 0.02082 | 0.506 | 0.01785 | 0.4543 |
| ***Change in Neuropathic Pain (between admission and discharge)*** | ***Coefficient (ß)*** | ***SE*** | ***P-value*** | ***R^2^*** | ***F-statistic*** |
| Model 2a (adjusted for rehabilitation time) |  |  |  |  |  |
| Intercept | -1.47169 | 2.01386 | 0.476 |  |  |
| Change in adaptation | -0.04190 | 0.04734 | 0.345 |  |  |
| Rehabilitation time | 0.01486 | 0.01974 | 0.463 | 0.08321 | 0.6808 |
| Model 2b (adjusted for rehabilitation time) |  |  |  |  |  |
| Intercept | 4.91976 | 12.92404 | 0.709 |  |  |
| Change in temporal summation of pain | 1.62712 | 1.58323 | 0.320 |  |  |
| Rehabilitation time | -0.06962 | 0.12550 | 0.587 | 0.07485 | 0.6068 |
| Model 2c (adjusted for rehabilitation time) |  |  |  |  |  |
| Intercept | -7.96901 | 9.69760 | 0.4241 |  |  |
| Change in modulation profile | -2.91774 | 1.18798 | 0.0267* |  |  |
| Rehabilitation time | 0.13544 | 0.09417 | 0.1709 | 0.3231 | 3.58 |
| ***Change in Musculoskeletal Pain (between admission and discharge)*** | ***Coefficient (ß)*** | ***SE*** | ***P-value*** | ***R^2^*** | ***F-statistic*** |
| Model 2a (adjusted for rehabilitation time) |  |  |  |  |  |
| Intercept | -8.72363 | 7.31691 | 0.254 |  |  |
| Change in adaptation | -0.88914 | 1.06265 | 0.418 |  |  |
| Rehabilitation time | 0.09629 | 0.07237 | 0.206 | 0.1603 | 1.241 |
| Model 2b (adjusted for rehabilitation time) |  |  |  |  |  |
| Intercept | -0.6934844 | 1.8903200 | 0.720 |  |  |
| Change in temporal summation of pain | 0.0177639 | 0.0423897 | 0.682 |  |  |
| Rehabilitation time | -0.0002548 | 0.0187642 | 0.989 | 0.01336 | 0.08799 |
| Model 2c (adjusted for rehabilitation time) |  |  |  |  |  |
| Intercept | -0.94059 | 1.84825 | 0.619 |  |  |
| Change in modulation profile | -0.03882 | 0.04130 | 0.364 |  |  |
| Rehabilitation time | 0.00362 | 0.01876 | 0.850 | 0.06367 | 0.442 |
